# RAGE and its ligand amyloid beta promote retinal ganglion cell loss following ischemia-reperfusion injury

**DOI:** 10.3389/fncel.2023.1156084

**Published:** 2023-04-12

**Authors:** Nafiseh Seyed Hosseini Fin, Dana Georgevsky, Maria B. Sukkar, S. Mojtaba Golzan

**Affiliations:** ^1^Vision Science Group, Graduate School of Health, University of Technology Sydney, Sydney, NSW, Australia; ^2^Pharmacy Discipline, Graduate School of Health, University of Technology Sydney, Sydney, NSW, Australia

**Keywords:** receptor for advanced glycation end products, amyloid beta, retinal ischemia-reperfusion, retinal ganglion cell loss, glaucoma

## Abstract

**Introduction:**

Glaucoma is a progressive neurodegenerative disease associated with age. Accumulation of amyloid-beta (Aß) proteins in the ganglion cell layer (GCL) and subsequent retinal ganglion cell (RGC) loss is an established pathological hallmark of the disease. The mechanism through which Aß provokes RGC loss remains unclear. The receptor for the advanced glycation end product (RAGE), and its ligand Aß, have been shown to mediate neuronal loss *via* internalizing Aß within the neurons. In this study, we investigated whether the RAGE–Aß axis plays a role in RGC loss in experimental glaucoma.

**Methods:**

Retinal ischemia was induced by an acute elevation of intraocular pressure in RAGE^–/–^ and wild-type (WT) control mice. In a subset of animals, oligomeric Aß was injected directly into the vitreous of both strains. RGC loss was assessed using histology and biochemical assays. Baseline and terminal positive scotopic threshold (pSTR) were also recorded.

**Results:**

Retinal ischemia resulted in 1.9-fold higher RGC loss in WT mice compared to RAGE^–/–^ mice (36 ± 3% *p* < 0.0001 vs. 19 ± 2%, *p* = 0.004). Intravitreal injection of oligomeric Aß resulted in 2.3-fold greater RGC loss in WT mice compared to RAGE^–/–^ mice, 7-days post-injection (55 ± 4% *p* = 0.008 vs. 24 ± 2%, *p* = 0.02). We also found a significant decline in the positive scotopic threshold response (pSTR) amplitude of WT mice compared to RAGE^–/–^ (36 ± 3% vs. 16 ± 6%).

**Discussion:**

RAGE^–/–^ mice are protected against RGC loss following retinal ischemia. Intravitreal injection of oligomeric Aß accelerated RGC loss in WT mice but not RAGE^–/–^. A co-localization of RAGE and Aß, suggests that RAGE–Aß binding may contribute to RGC loss.

## Introduction

Glaucoma, classified as a neurodegenerative disease (Gupta and Yücel, 2007), is associated with increased intraocular pressure (IOP) and subsequent retinal ganglion cell (RGC) loss. Current evidence suggests that a significant proportion of RGC loss occurs before a clinical diagnosis is made, predisposing an individual to vision loss (Kerrigan-Baumrind et al., 2000). As a result, recent studies have focused on identifying underlying mechanisms involved in RGC loss, and strategies that may promote RGC survival in glaucoma. RGC loss due to activation of molecular pathways associated with glial activation (Bosco et al., 2008), mitochondrial dysfunction (Kong et al., 2009), oxidative stress (Saccà and Izzotti, 2008), neurotrophic factors (Gupta et al., 2014), and release of tumor necrosis factor (Nakazawa et al., 2006) have previously been reported. There is evidence indicating that the receptor for advanced glycation end products (RAGE) may also play a role in glaucoma pathogenesis (Tezel et al., 2007). RAGE binds to a diverse repertoire of endogenous ligands including advanced glycation end products (AGEs), amyloid-beta (Aß), High Mobility Group Box 1 (HMGB1), and members of the S100 protein family (Yan et al., 1996; Hofmann et al., 1999; Ramasamy et al., 2011; Meneghini et al., 2013). RAGE and its ligands are implicated in various chronic diseases associated with aging, particularly neurodegenerative diseases such as Alzheimer’s disease (AD) (Criscuolo et al., 2017), and are known to be involved in underlying processes associated with neuronal loss.

Amongst RAGE ligands, Aβ has been shown to have a potent neurotoxic effect on RGCs (Guo et al., 2007) and has been identified within the ganglion cell layer (GCL) in ocular tissues collected from glaucoma patients (Gupta et al., 2014). There is evidence that RAGE upregulation actively mediates the neurotoxic effects of Aβ through transporting the ligand across the blood-brain barrier into the brain *via* the transcytosis (Deane et al., 2009; Takuma et al., 2009). While, the role of RAGE–Aß activation in neuronal loss during neurodegeneration (e.g., AD) has been widely investigated (Yan et al., 1995, 1996), little is known about its role in glaucoma. RAGE and Aß have been detected in the GCL following the retinal ischemia (Guo et al., 2007; Mi et al., 2012). Mi et al. (2012) showed that upregulation of RAGE and Aß leads to RGC loss in experimental glaucoma. An earlier study by Guo et al. (2007) showed that targeting Aß could reduce RGC loss in a mouse model of an experimental glaucoma. Consistent with these findings, increased RAGE levels have been reported in the optic nerve head of donor ocular tissues with recorded glaucomatous history (Tezel et al., 2007). These findings suggest that both RAGE and Aß play a role in the underlying mechanisms of RGC loss in glaucoma.

Several experimental models have been developed to study RGC loss associated with the glaucoma (Goldblum and Mittag, 2002; Johnson and Tomarev, 2010). Amongst many, the retinal ischemia-reperfusion (IR) injury model, as a result of acute ocular hypertension (AOH), provides a significant insight into the neurodegenerative damage within the GCL. The pathophysiological process associated with this model is based on disrupting the normal blood flow in and out of the retina which ultimately leads to accelerated RGC loss. Using the IR injury model, we aimed to establish the potential role of RAGE and Aß in mediating RGC loss following AOH. Furthermore, we investigated whether exogenous injection of Aß into the vitreous would lead to RAGE–Aß activation and subsequent RGC loss. To implement our hypothesis, we used a mouse line in which the gene for RAGE (*Ager*- receptor for Advanced Glycation End product) was deleted, producing the RAGE^–/–^ line. RAGE^–/–^ mice show significantly higher activity in darkness, and higher sensitivity to auditory signal. Furthermore, deletion of RAGE have shown to have minimal effects on the animal’s spatial learning ability (Sakatani et al., 2009). Collectively, the model does not alter the underlying retinal pathophysiology and thus, provides a sound platform to address the aims of the present study.

## Materials and methods

### Animals

Male RAGE deficient (RAGE^–/–^, C57BL/6 mice backcrossed >20 generations into C57BL/6J) mice (aged 10–12 weeks, 22 ± 3 g) and aged-matched, independent male C57BL/6 wild type (WT) control mice (23 ± 3 g) were used in this study. C57BL/6 WT animals were purchased from the Animal Resources Centre (Perth, Australia). RAGE^–/–^ mice were kindly provided by Professor Ann Marie Schmidt (New York University Langone Medical Centre, New York, NY, USA). All animals were kept on a cyclic light (12 h on; 12 h off; ∼300 lx), air-conditioned room (21 ± 2°C) with water and food available *ad libitum*. The study was approved by the Animal Care and Ethics Committee, University of Technology Sydney (ETH17-0549). All procedures involving animals were conducted in accordance with the Australian code for the care and use of animals for scientific purposes and the guidelines of the Association for Research in Vision and Ophthalmology statement for the use of animals in vision research.

### Study groups

Animals were randomly assigned to one of the two following study groups.

#### Group 1- retinal ischemia/reperfusion injury

To determine the potential role of RAGE in RGC loss in glaucoma, we hypothesized that if RAGE upregulation is indeed involved in the activation of signaling pathways that leads to RGC loss, then RAGE^–/–^ mice would be protected against RGC loss following retina ischemia. To investigate this, we followed the experimental procedure described by Hartsock et al. (2016) to induce RGC loss as a result of retinal ischemia following AOH. Briefly, RAGE^–/–^ (*n* = 12) and WT control (*n* = 12) mice were anesthetized with isoflurane (induced with 5% isoflurane in 2.5 L/min oxygen and maintained at 2% isoflurane in 2.5 L/min oxygen) and placed on a heating mat under a surgical microscope. A drop of 1% proparacaine hydrochloride topical anesthesia was instilled (Alcon, Fort Worth, TX, USA), followed by 1% tropicamide (Alcon) for pupil dilation. The anterior chamber of the right eye was cannulated using a 33-gauge needle (Precise Medical Supplies, Banksmeadow, NSW, Australia) and connected to an elevated reservoir of sterile balanced salt solution placed 120 cm above the animal to generate a pressure over 90 mmHg. Elevated IOP was maintained for 45 min, after which the IOP was returned to normal levels by allowing reperfusion (Jia et al., 2017). A drop of the antibiotic Chlorsig 0.5% (Aspen Pharmacare, St Leonards, NSW, Australia) and a single subcutaneous dose of the analgesic carprofen (5 mg/kg) (Zoetis, Rhodes, NSW, Australia) were also administered before animal recovery. A successful elevation of IOP was confirmed visually by the presence of ocular distention and the absence of leakage. The other eye was injected but not pressurized and was used as a control. Baseline and terminal Electroretinogram (ERG) were also performed. All animals were euthanized 7 days post-procedure and tissues were collected for histology and *in vitro* assays (details below).

#### Group 2- exogenous intravitreal injection of Aß

To determine the potential role of RAGE–Aß activation in RGC loss in glaucoma, we hypothesized that RAGE^–/–^ mice would be protected against RGC loss following the exogenous injection of Aß into the vitreous. To address this, we injected oligomerized Aß_1–42_ directly into the vitreous of RAGE^–/–^ (*n* = 12) and WT (*n* = 12) mice. While RAGE is known to bind monomeric, oligomeric, and even fibrillar forms of Aß at the neuronal cell surface (Takuma et al., 2009), however, previous studies have shown that oligomeric Aß has the most potent neurotoxic effect on RGCs (Guo et al., 2007). Hence, we decided to use the oligomeric isoform of Aß.

To produce oligomeric Aß, we used a protocol described previously by Fa et al. (2010). Briefly, 1 mg of lyophilized Aß_1–42_ peptide (Bachem AG, Bubendorf, Switzerland) was resuspended in ice-cold 1,1,1,3,3,3-hexafluoro-2-propanol (HFIP) (Sigma, Macquarie Park, NSW, Australia) to obtain a 1 mM solution, vortexed for 30 s and immediately aliquoted into three polypropylene Eppendorf tubes. The Eppendorf tubes were incubated for 2 h at room temperature to allow for Aß monomerization. Next, the Aß_1–42_–HFIP solution was concentrated using a SpeedVac centrifuge (800 *g* at room temperature). The prepared solution was then resuspended in 5% dimethyl sulfoxide (DMSO) anhydrous in 0.1 M phosphate-buffered saline (PBS) to obtain a final concentration of 5 mM Aß_1–42_. Finally, the samples were sonicated in a water bath for 10 min to ensure complete resuspension. Twenty-four hours before the injection, 5 mM Aß_1–42_–DMSO aliquots were resuspended in PBS to make a final Aß_1–42_ solution of 2.3 mg/2 mL (Guo et al., 2007).

For intravitreal injection, animals were anesthetized with a mixture of ketamine (75 mg/kg) and xylazine (10 mg/kg) and placed on a heat mat under a surgical microscope. A drop of proparacaine hydrochloride 1% was instilled for topical eye anesthesia. A micromanipulator was used to hold a 10 μL Hamilton syringe prefilled with the Aß solution and connected to a 33-gauge needle. A single dose containing 2 μL of Aß solution was then injected into the vitreous at a 45-degree angle through the sclera. A drop of the antibiotic Chlorsig 0.5% and a single subcutaneous dose of the analgesic carprofen (5 mg/kg) was also administered before animal recovery. A single dose of 2 μL vehicle was injected into the other eye. Baseline and terminal ERG were also performed. Animals were split into two subgroups and euthanized at two time points; 72 h (*n* = 6 from each strain) and 7 days (*n* = 6 from each strain) post-Aß injection. These time points were chosen based on a previous study that showed maximum potency of Aß on RGCs (Guo et al., 2007).

### Electroretinogram

The most sensitive response in the dark-adapted ERG is known as scotopic threshold response (STR), which originates from the inner retina, and specifically RGCs. STRs were obtained in a dark room using a dim red light from dark-adapted mice (Ocuscience, Henderson, NV, USA). Mice were anesthetized using a combination of ketamine (75 mg/kg) and xylazine (10 mg/kg) and placed on a heating pad. Pupils were dilated with 1% tropicamide. Contact lens-embedded thread electrodes were placed on each cornea and moisturized using 0.3% lubricating eye gel. Two stainless steel reference electrodes were inserted subcutaneously on each cheek. A third electrode was used as the ground reference and was inserted subcutaneously into the tail skin. To measure the STR, responses from 30 flashes (3 cd s m–2) with 2 s intervals were averaged. A positive STR (pSTR) was measured from the baseline to maximum peaks of the waveform at the flash intensity.

### *In vitro* assays

#### Sample processing

For protein quantification using immunoblotting, the retina was dissected and immediately frozen using carbon dioxide. For histology and immunostaining, whole eyes were enucleated, submerged into 4% paraformaldehyde, and stored overnight at 4°C. Eyes were then transferred to a tissue processor (Thermo Fisher, Scoresby, VIC, Australia) and were processed in subsequent cycles of different reagents (ethanol and xylene) for dehydration, subjected to wax infiltration and finally embedded in paraffin wax in sagittal orientation. Tissues were then sectioned at 10 um thickness using a microtome (Thermo Fisher) and placed on SuperFrost slides (Thermo Fisher) for further analysis.

### Hematoxylin and Eosin (H&E) staining

All slides were heated for deparaffinization at 65°C for 30 min before staining. The slides were rehydrated with xylene and 100% ethanol in 5-min intervals and stained with filtered 0.1% hematoxylin for 5 min. The slides were then washed with running water, submerged in 0.5% eosin (1.5 g dissolved in 300 mL of 95% ethanol) for 5 min, and washed in a graded series of ethanol.

### Histology staining and imaging

Tissue sections were permeabilized in 1% Triton X-100 in PBS for 30 min at room temperature. After rinsing in PBS, the samples were blocked with 1% bovine serum albumin in PBS for 30 min. The slides were then labeled with the relevant primary antibody (Table 1) and incubated at 4°C overnight. The slides were labeled with the appropriate secondary antibody (Table 2) for 2 h and then washed and labeled with a diamidino-2-phenylindole (DAPI) mounting medium. Cover-slipped slides were imaged using a BX51 upright EPI fluorescence microscope (Olympus, Tokyo, Japan). Thioflavin S staining was performed after immunostaining to co-localize Aß deposits with other antigens used in our experiments. Following immunostaining, slides were stained with 1% thioflavin S in 80% ethanol for 12 min at room temperature. Slides were then washed in 80% and 95% ethanol and PBS, cover-slipped, and imaged at 460 nm excitation using a BX51 upright EPI fluorescence microscope (Olympus). Radial cross-sectional images from the optic nerve head (experimental and control eye) were used to manually quantify RGC numbers in 200 μm increments of the GCL.

**TABLE 1:** List of primary antibodies.

Primary antibody (dilution)	Target	Supplier	RRID
Brn3a (1:1,000) for immunostaining and (1:500) for immunoblotting	Rabbit polyclonal antibody	Sigma, B9684	AB_476814
Aß (1:100) (for immunostaining)	Rabbit monoclonal antibody	Cell Signaling Technology, MA, USA, D54D2	AB_2797642
Caspase 3 (1:1,000)	Rabbit polyclonal antibody	Novus Biologicals, CO, USA, NB100-56112	AB_837848
RAGE (1:1000)	Rabbit polyclonal antibody	Sigma, R5278	AB_477463
Aß (1:1,000) (for immunoblotting)	Mouse monoclonal antibody	Life Technologies, VIC, Australia, 13-0100Z	AB_2532992
GAPDH (1:1,000)	Chicken polyclonal antibody	Millipore, MA, USA, AB2302	AB_10615768

**TABLE 2:** List of secondary antibodies.

Secondary antibody (dilution)	Target	Supplier	RRID
IRDye 800CW (0.5:2,000) (for immunoblotting)	Chicken polyclonal antibody	Millennium Science, VIC, Australia, LCR-925-32218	AB_1850023
IRDye 800CW (0.5:2,000) (for immunoblotting)	Mouse monoclonal antibody;	Millennium Science, LCR-925-32210	ABB_621842
IRDye 680RD (0.5:2,000) (for immunoblotting)	Rabbit polyclonal antibody; used in western blotting.	Millennium Science, LCR-925-68071	AB_10956166
Cy3 (1:1,000) (for immunostaining)	Rabbit polyclonal antibody; used in immunostaining.	Jackson ImmunoResearch, PA, USA, 111-165-003	AB_2338000

### Immunoblotting

To quantify Brn3a, Aß, and caspase 3, retinas were homogenized with a micro-pestle in lysis buffer (MCL1-1KT, Sigma Aldrich, Castle Hill, NSW, Australia) and centrifuged at 10–15,000 rpm for 10 min. The protein concentration was measured in the supernatant using a protein microassay kit (Life Technologies, Mulgrave, VIC, Australia). Ten micrograms of each sample was loaded and separated by size in a Novex 4–20% Tris-glycine gel (Life Technologies). Proteins were then transferred to a polyvinylidene difluoride (PVDF) membrane using an iBlot device (Life Technologies) and incubated in relevant primary and secondary antibodies (Tables 1, 2) using the iBind incubation device (Life Technologies). Following incubation for 2.5–3 h, blots were visualized and imaged using an Odyssey imager (Licor, Lincoln, NE, USA) and analyzed using ImageJ. For densitometry analysis, briefly, a rectangular area around each band was selected, and the software gave histograms indicating the intensity of each band. ImageJ was used to quantify color intensity. To calculate changes in each band, the relevant intensity value of each protein was divided by the reference intensity value of glyceraldehyde 3-phosphate dehydrogenase (GAPDH).

### Statistical analysis

All statistical tests were performed using GraphPad Prism 7 (GraphPad Software Inc., San Diego, CA, USA). The data are presented as mean ± standard error of the mean (SEM). Unpaired *t*-test was used to compare groups. Statistical differences were considered to be significant at *p*-values < 0.05.

## Results

### RAGE^–/–^ mice are protected against RGC loss induced by ischemia

**FIGURE 1:**
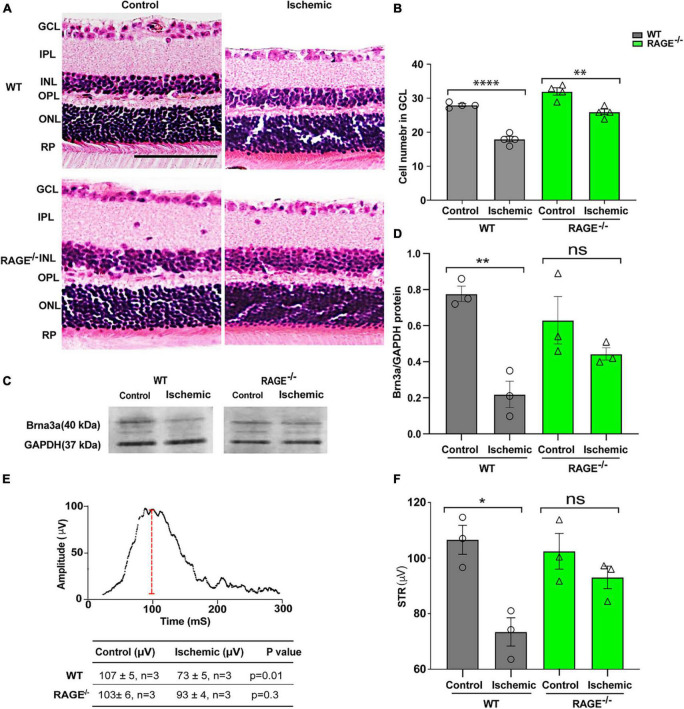
RAGE^–/–^ mice are protected against RGC loss and inner retinal dysfunction following ischemia. **(A)** H&E staining of WT and RAGE^–/–^ retinal sections. **(B)** Comparison of the alterations of RGC numbers in WT and RAGE^–/–^ mice after ischemia. A significant decline in RGC numbers was observed in both WT and RAGE^–/–^ mice (*n* = 4). **(C)** Western blotting of WT and RAGE^–/–^ mice using Brn3a antibody showing the Brn3a band around 40 kDa. GAPDH was used as a control. **(D)** Densitometric analysis of Brn3a/GAPDH fold change showed a significant Brn3a downregulation in WT ischemic mice compared with WT control, but not in RAGE^–/–^ groups. **(E,F)** Alterations of pSTR in WT and RAGE^–/–^ mice after AOH. Graph representing the comparison of the pSTR decline in WT and RAGE^–/–^ mice after AOH. *p = 0.01, **p = 0.003, ****p < 0.0001, **p = 0.004, ns: no significance, unpaired *t*-test. IPL, inner plexiform layer; INL, inner nuclear layer; ONL, outer nuclear layer, PR, photoreceptor. The scale bar is 100 μm.

We investigated the impact of retinal ischemia induced by ischemia on RGC loss in WT and RAGE^–/–^ mice. In WT mice, retinal ischemia led to a significant decrease in both the number of RGCs (Figures 1A, B) and Brn3a + cell density and protein expression, a specific molecular marker of RGC cell survival (Figures 1C, D and Supplementary Figures 1A, B). It also led to a significant decrease in the amplitude of the pSTR, indicating that RGC loss was associated with a concomitant loss in the RGC function (Figures 1E, F). Notably, however, while induction of ischemia in RAGE^–/–^ mice caused a significant decrease in RGC number using H&E staining (Figures 1A, B), the Brn3a + cell counting exhibited a non-significant reduction of RGCs in GCL (Supplementary Figure 1B). Further, the magnitude of RGC loss was 09-fold lower than that observed in WT mice (Figure 1B). The relatively smaller extent of RGC loss demonstrated by Brn3a + cell counting in RAGE^–/–^ mice was accompanied by a non-significant decrease in Brn3a protein abundance (Figures 1C, D) and had no significant impact on RGC function (Figures 1E, F) indicating that RAGE^–/–^ mice are largely protected against ischemic-related RGC loss.

### RAGE^–/–^ mice have reduced levels of Aß protein in the GCL following induction of retinal ischemia

**FIGURE 2:**
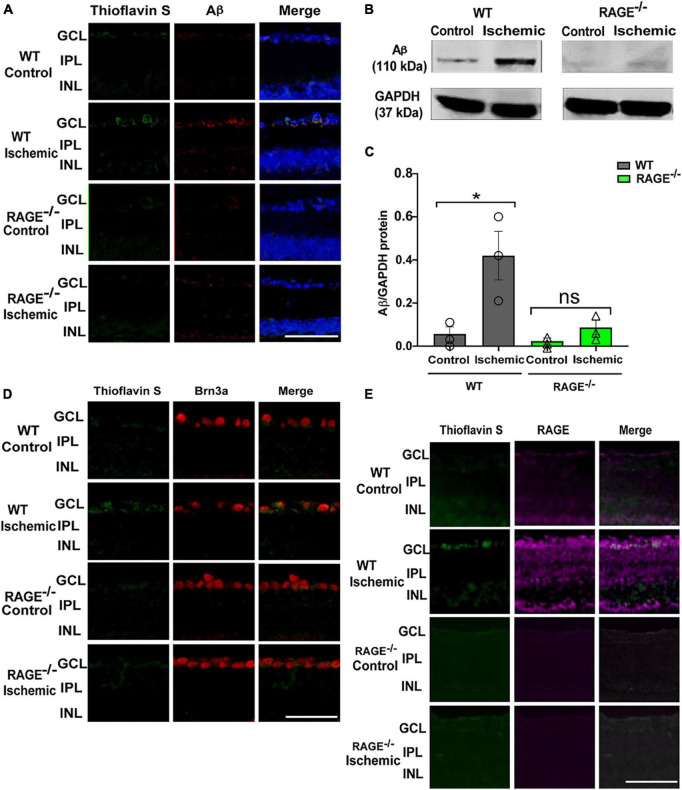
Elevation of Aß deposition and RAGE upregulation in WT ischemic mice. **(A)** Immunostaining of WT and RAGE^–/–^retinas using Thioflavin S staining (green), Aß antibody (red), DAPI (blue) (*n* = 3). Aß deposition was detected in GCL of WT ischemic mice. **(B)** Western blotting of Aß antibody in WT and RAGE^–/–^ retinas shows the Aß band at 110 kDa. GAPDH was used as a control. **(C)** Densitometric analysis of Aß alteration after ischemia in WT and RAGE^–/–^. Data is presented as Aß/GAPDH fold change (*n* = 3). **(D)** An increased labeling of Aß (green) and Brn3a (red) was detected in the GCL of WT ischemic mice (*n* = 3). **(E)** Immunostaining of ischemic and control WT and RAGE^–/–^ retinas using RAGE antibody (magenta). Increased labeling of Thioflavin S and RAGE in the GCL of ischemic WT mice is observed. *p = 0.04, ns: no significance, unpaired *t*-test. The scale bar is 50 μm.

To determine whether protection against RGC loss in RAGE^–/–^ mice was associated with a concomitant decrease in the accumulation of RAGE ligands in the retinal tissue, we examined Aß protein levels following retinal ischemia in WT and RAGE^–/–^ mice. Using Thioflavin S staining and immunostaining the location of Aß deposition within the retinal tissue in GCL of WT ischemic mice but not RAGE^–/–^ mice was confirmed (Figure 2A). Evaluation of Aß protein expression using immunoblotting demonstrated a significant increase in the abundance of Aß protein in WT but not in RAGE^–/–^ mice (Figures 2B, C). To determine whether increased Aß deposition was associated with RGC loss, double immunolabeling using Aß and Brn3a antibodies was done in retina sections. Outcomes of this analysis revealed significant up-regulation of Aß protein and its co-localization with Brn3a protein, within the GCL, in WT but not RAGE^–/–^ mice (Figure 2D). Moreover, in WT mice, AOH induced significant up-regulation in RAGE receptor expression within the GCL (Figure 2E). Taken together, these findings suggest that AOH leads to increased deposition of Aß and RAGE protein within the GCL which in turn may promote RGC loss *via* activation of the RAGE signaling axis.

### Intravitreal injection of oligomeric Aß promotes RGC loss in WT but not RAGE^–/–^ mice

**FIGURE 3:**
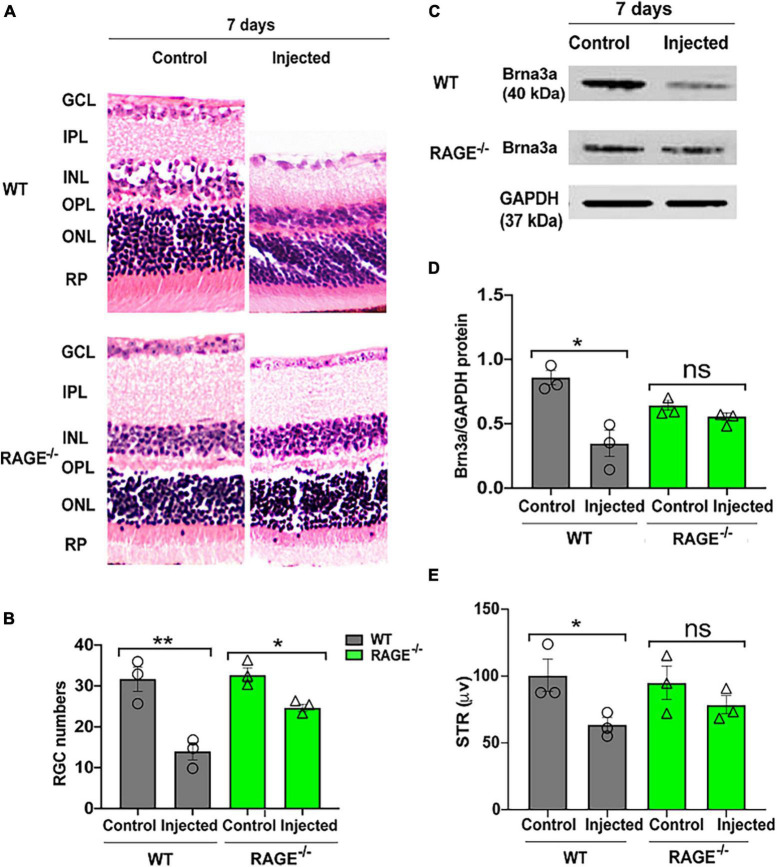
Intravitreal injection of Aß exacerbates RGC loss and inner retinal dysfunction in WT and not RAGE^–/–^ mice. **(A)** H&E staining of WT and RAGE^–/–^ retina, 7 days after Aß injection (*n* = 3). Significant RGC loss in both WT and RAGE^–/–^ retina. RGCs were measured manually around the optic nerve head in increments of 300 μm. **(B)** Comparison of the alterations of RGC numbers in WT and RAGE^–/–^ mice after Aß injection. Using H&E staining, a significant decline in RGC numbers was observed in both WT and RAGE^–/–^ mice (*n* = 3). **(C)** Western blotting of WT and RAGE^–/–^ retinas after intravitreal injection of Aß. GAPDH was used as a control (*n* = 3). **(D)** A significant Brn3a downregulation was observed in WT, 7 days after Aß injection. Brn3a downregulation in Aß injected eyes of each mouse was normalized as Brn3a/GAPDH (*n* = 3). **(E)** Graph representing pSTR analysis of WT and RAGE^–/–^ (*n* = 3). *p = 0.02, **p = 0.008, *p = 0.04, *p = 0.01, ns: no significance, unpaired *t*-test. The scale bar is 100 μm.

Given our observations above, we investigated whether (i) intravitreal injection of oligomeric Aß promotes RGC loss in WT mice and (ii) RAGE^–/–^ mice are afforded protection against Aß-mediated RGC loss. Accordingly, we quantified RGC numbers, Brn3a protein expression and changes in pSTR 72 h and 7 days following intravitreal Aß injection in WT and RAGE^–/–^ mice. There was no significant change in RGC numbers, Brn3a protein abundance or pSTR amplitude 72 h post-intravitreal Aß injection in WT mice (Supplementary Figure 2). However, we observed a significant decrease in RGC numbers together with a concomitant decrease in Brn3a protein abundance and significant repression of pSTR, 7 days post-intravitreal Aß in WT mice (Figures 3A–E and Supplementary Figure 1C). In contrast to findings in WT mice, intravitreal Aß had no significant effect on Brn3a + cell density and protein expression or pSTR 7 days post-injection in RAGE^–/–^ mice (Figures 3C–E and Supplementary Figure 1C).

**FIGURE 4:**
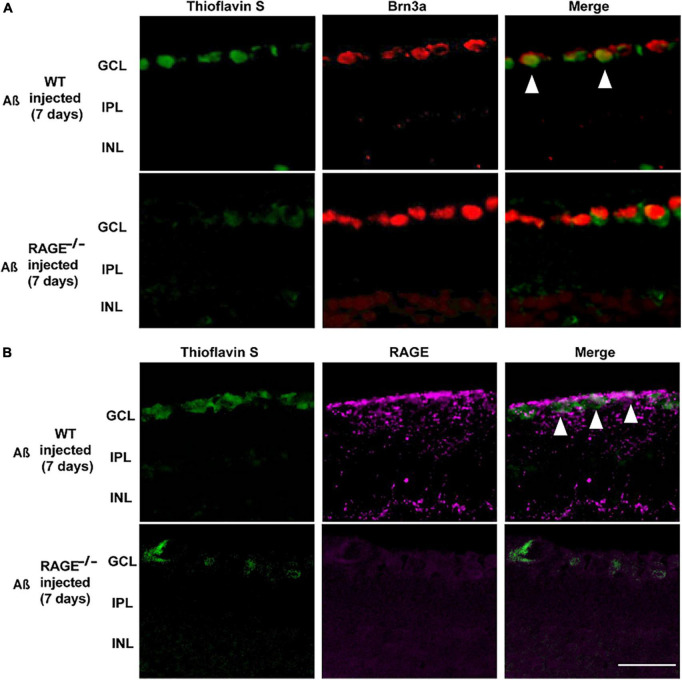
Increased labeling of Brn3a, RAGE, and Thioflavin S were identified in the retina following Aß injection. **(A)** Immunostaining of WT and RAGE^–/–^ mice. Brn3a (red) was used to label RGCs. Thioflavin S (green) was used to stain Aß. Increased labeling of Brn3a and Thioflavin S in the WT retina was observed 7 days after Aß injection (*n* = 3). **(B)** RAGE upregulation (magenta) was observed in the GCL of WT mice, 7 days after Aß injection. Increased labeling of thioflavin S and RAGE was observed in the GCL of WT retina, 7 days after Aß injection (*n* = 3). The scale bar is 50 μm.

Consistent with these findings, there was evidence of increased Aß Brn3a positive expression within the same region of the GCL, 7 days post-intravitreal Aß injection in WT mice (Figure 4A). Increased expression of Aß deposits with RAGE in the GCL provided further evidence for a direct role for Aß in RAGE-dependent RGC loss (Figure 4B).

### RAGE-dependent RGC loss is mediated *via* apoptotic cell death

**FIGURE 5:**
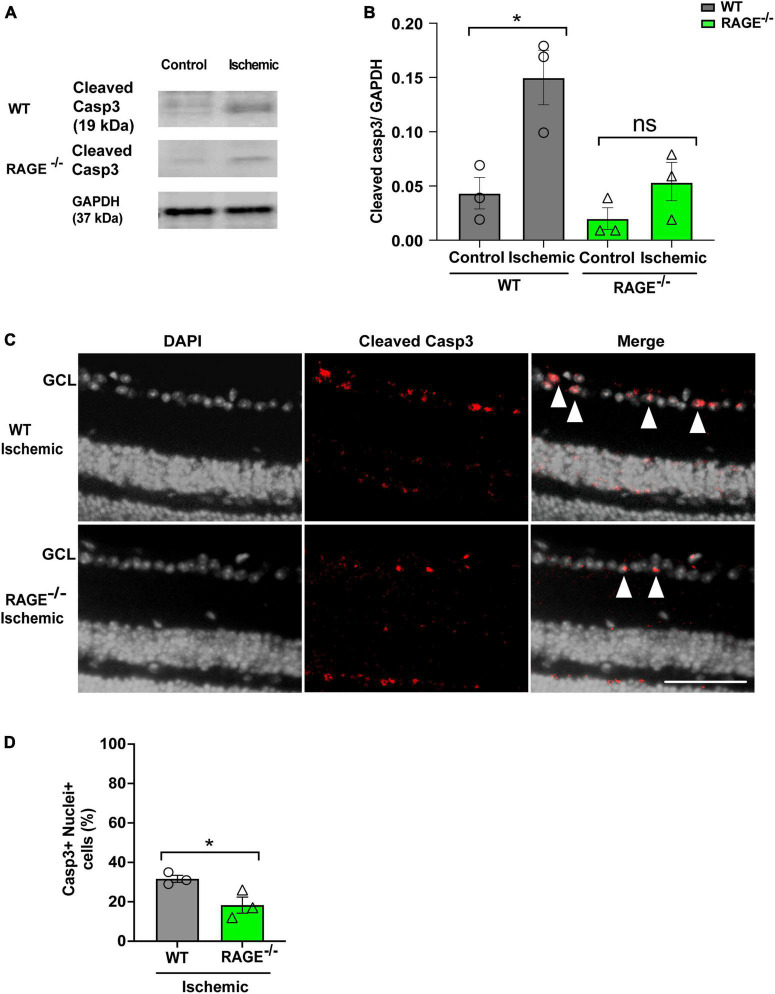
Assessment of apoptotic cell death in the ischemic group. **(A,B)** Western blotting of cleaved caspase 3 (19kDa) in control and ischemic retinas of WT and RAGE^–/–^ mice. GAPDH was used as a control, followed by quantification of cleaved casp3 in animal groups using densitometry (*n* = 3). **(C)** Immunostaining shows a co-staining of cleaved casp3 (red) and the nuclei (gray) in the GCL of ischemic WT and RAGE^–/–^ groups (*n* = 3). **(D)** Quantification represents a significant increase in the density of casp3 + nuclei + cells in the WT ischemic group, compared to RAGE^–/–^ mice. *p = 0.02, *p = 0.04, ns: no significance unpaired *t*-test. The scale bar is 100 μm.

**FIGURE 6:**
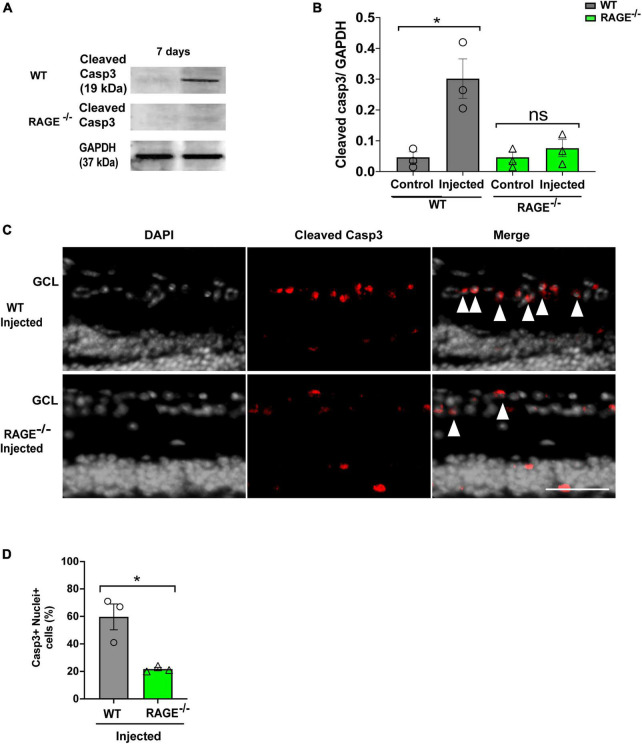
Assessment of apoptotic cell death in the Aβ injected group. **(A,B)** Western blotting of cleaved caspase 3 (19kDa) in control and injected retinas of WT and RAGE^–/–^ mice. GAPDH was used as a control, followed by quantification of cleaved casp3 in animal groups using densitometry (*n* = 3). **(C)** Immunostaining shows a increased labeling of cleaved casp3 (red) and the nuclei (gray) in the GCL of injected WT and RAGE^–/–^ groups (*n* = 3). **(D)** Quantification represents a significant increase in the density of casp3 + nuclei + cells in WT injected group, compared to RAGE^–/–^ mice. *p = 0.02, ns: no significance unpaired *t*-test. The scale bar is 100 μm.

We used two separate but complementary approaches to establish a role for the Aß-RAGE signaling axis in ischemic-related RGC loss. Thus, we measured protein levels of cleaved caspase-3 (casp3), a marker of cellular apoptosis, to confirm that RGC loss occurred *via* programmed cell death in each model (Figures 5, 6). Indeed, in WT mice, RGC loss induced by ischemia was associated with a significant increase in cleaved casp3 expression in the retinal tissue (Figures 5A, B). These findings were confirmed by immunostaining of cleaved casp3 in the retina of the WT ischemic group (Figures 5C, D). Likewise, intravitreal Aß injection led to a significant RGC loss due to the apoptosis in the WT group as revealed by cleaved casp3 immunoblotting and immunostaining (Figures 6A–D). On the other hand, ischemia and intravitreal Aß injection did not lead to a significant increase in cleaved casp3 in RAGE^–/–^ mice, consistent with protection against RGC loss in these mice (Figures 5A–D).

## Discussion

In this study, we investigated the potential role of RAGE and its ligand Aß in mediating RGC loss in experimental glaucoma. We found that RAGE deficient mice are protected against RGC loss when exposed to an acute elevation of intraocular pressure to induce retinal ischemia. This confirms our hypothesis that RAGE plays a role in RGC loss in glaucomatous-type pathology. Retinal ischemia also resulted in an increase in Aß levels in the GCL of ischemic WT retinas (but not RAGE^–/–^), confirming the role of Aß in RGC loss, as reported previously by Guo et al. (2007). To further confirm that RAGE mediates the neurotoxicity effects of Aß in RGCs, we injected exogenous oligomeric Aß directly into the vitreous of WT and RAGE^–/–^ mice. RGC loss rate was significantly higher in the WT mice compared with RAGE^–/–^ mice, 7 days post-Aß injection. Double staining for RGC/Aß and RAGE/Aß also confirmed a co-localization of all these three markers in the GCL of WT mice, suggesting a potential RAGE–Aß binding that ultimately leads to RGC loss.

Recent studies have established the role of RAGE in neurodegeneration, mainly AD (Derk et al., 2018). In AD, higher levels of RAGE have been identified in endothelial cells, neurons, and microglia surrounding amyloid plaques (Yan et al., 1996). In brain endothelium, the influx of circulating Aß across the blood-brain barrier into the brain is enhanced by RAGE receptor *via* transcytosis (Deane et al., 2009), and within the central nervous system, RAGE has been shown to internalize Aβ oligomers into the neuronal and glial cells *via* endocytosis (Mohamed and Posse de Chaves, 2011). While similar to AD, glaucoma is classified as a neurodegenerative disease (Artero-Castro et al., 2020), the mechanism in which RAGE mediates RGC loss in glaucoma, is unclear. Within the retina, RAGE is expressed in a number of cells including muller cells, retinal pigment epithelium cells, and RGCs (Howes et al., 2004; Kaji et al., 2007; Tezel et al., 2007). In glaucomatous eye tissues, RAGE has been detected in the GCL with higher levels detected on glial cells and Muller cells (Tezel et al., 2007). Consistent with this, we observed an increase in RAGE expression in the GCL following ischemia, compared with their fellow control eyes.

Receptor for the advanced glycation end product-ligand activation has been linked to glaucomatous pathology observed in the ischemic retina (Mi et al., 2012). Increased HMGB1 levels, a RAGE ligand, has been shown to induce an inflammatory response, contributing to neurotoxicity *via* RAGE signaling, 7 days post retinal ischemia (Dvoriantchikova et al., 2011). Lycium Barbarum Polysaccharide has been shown to protect against Aß mediated neurotoxicity of RGC, blood-retinal barrier, and blood vessels during retinal ischemia *via* down-regulation of RAGE (Mi et al., 2012). This can be explained by the fact that RAGE and Aß are transferred from the blood circulation through the vascular RAGE up-regulation (Mi et al., 2012). Tezel et al. (2007) also reported an upregulation of RAGE and its ligand AGE in the glaucoma. Amongst the various RAGE ligands, we focused on Aß in this study as there is mounting evidence demonstrating its neurotoxic role in the glaucoma (Guo et al., 2007).

Amyloid-beta is produced by the consecutive cleavage of amyloid-beta precursor protein (APP), which is expressed in RGCs of the retina in the human, mouse, rat, monkey, and rabbit (Morin et al., 1993; Löffler et al., 1995; Wang et al., 2011). While retinal APP is known to play a physiological role as a trophic factor in the neurodevelopment (Dinet et al., 2011; Dawkins and Small, 2014), enzymes that contribute to the amyloidogenic processing of APP and subsequent Aß aggregation, have been detected in the retina (Johnson et al., 2002; Yoshida et al., 2005; Prakasam et al., 2010). Retinal neurons are capable of switching APP processing to the amyloidogenic pathway under cellular stress such as ischemia (McKinnon et al., 2002; Liu et al., 2018). We observed a strong Aß signal in the GCL of WT mice following ischemia. Double staining for RAGE and Aß also showed co-localization in the GCL of ischemic retinas but not control retinas. This suggests that RAGE might be involved in transporting extracellular Aß into RGCs from the vitreous *via* endocytosis. Lana et al. (2017) showed that internalization of Aß was reduced by up to 55% following the RAGE blockage. However, our results showed that Aß internalization was not completely inhibited, suggesting that other pathways are involved in Aß translocation. Taken together, these findings suggest that RAGE regulates at least one of the pathways associated with Aß endocytosis, however, further studies are required to confirm this.

To study whether Aß alone mediates RGC loss, oligomeric Aß, which is not present in normal physiological conditions, was injected into the vitreous. In its oligomeric form, Aß is a highly penetrative molecule because of its amphipathic structure (Jin et al., 2016). Oligomeric Aβ is also capable of binding to RAGE (Jin et al., 2016). Our results confirm that RAGE mediates extracellular uptake of Aß as higher intracellular Aß expression was detected within RGC neurons in WT mice compared with RAGE^–/–^ mice. RAGE–Aß activation not only takes part in Aß endocytosis but also activates signaling pathways such as p38 MAPK (Takuma et al., 2009). Activation of MAPK pathways has been shown to be one molecular mechanism involved in RGC apoptosis in the glaucoma (Levkovitch-Verbin, 2015). Interestingly, Kheiri et al. (2018) reported that targeting the p38 MAPK pathway can efficiently suppress the neurotoxicity of Aß in AD. This confirms that RGC loss does mimic cerebral neuronal loss in AD and that inhibiting RAGE–Aß binding may be a useful and effective way to protect RGCs in glaucoma. Unfortunately, we did not investigate whether a similar signaling pathway such as p38 MAPK mediates RGC loss, and further studies are required to investigate this notion. However, we observed a cleaved caspase 3 upregulation in the GCL following Aß injection. These results suggest that RAGE–Aß is involved in RGC apoptosis, most likely *via* the activation of caspase cascades. The compilation of these findings suggests that the internalization of Aß within RGCs through RAGE–Aß activation (as explained earlier) initiates a cascade of events that ultimately leads to RGC loss *via* apoptosis. It is also likely that RAGE–Aß-mediated signaling pathways activate apoptosis *via* upregulation of intermediates such as nuclear factor-κB leading to caspase 3 activation and, eventually, to RGC loss. However, further investigations are needed to confirm this hypothesis.

Our study has a few limitations. The broader limitation of our study is the small sample size in each animal group and the inclusion of only male mice. The use of single gender animals was to remove any possible cofounding effect of gender differences on our findings. Other specific limitations of our study are as follows. First, this study was based on an acute model, and further studies, particularly using chronic models and human tissues should be completed prior to conceiving RAGE as a potential pharmacological target. While this acute model differs from chronic conditions in the timeline and etiological origin, nonetheless, it provides a sound platform for evaluating the underlying causes of retinal neurodegeneration in glaucoma. Second, the present study demonstrated that the RAGE–Aß mediates RGC loss *via* the apoptotic pathway, however, the underlying mechanistic link between RAGE-mediated signaling pathways and Aß neurotoxicity needs further investigation. Previous studies have shown that RAGE–Aß binding activates p38 MAPK, leading to intracellular accumulation and ultimately, Aß induced neuronal loss in the brain. The MAP kinases are indeed proposed as a primary candidate for RAGE-mediated signal transduction with both p38 and JNK implicated in the neurotoxic effects of Aß. By using selective inhibitors of JNK and p38 MAPK, it would be interesting to study the downstream effects of Aß on RGCs in glaucoma. Finally, complementary tests including *in vivo* behavioral studies such as orientation tests (Hetherington et al., 2000), maze tests (Coffey et al., 2002), and optokinetic tests (Schmucker et al., 2005) which measure visual performance in eyes may also be useful for analyzing retinal dysfunction.

## Conclusion

In conclusion, our results demonstrate, for the first time, how RAGE- Aß binding can amplify the damaging effects of Aß on RGC loss potentially *via* the apoptotic pathway. Our results also demonstrate that RAGE expression increases and remains elevated as long as Aß is present. This could lead to the production of a positive feedback loop through which RAGE initiates and perpetuates Aß neuronal toxicity. Collectively, these results suggest that RAGE plays an active role in Aß-driven cytotoxicity in RGCs.

## Data availability statement

The raw data supporting the conclusions of this article will be made available by the authors, without undue reservation.

## Ethics statement

This animal study was reviewed and approved by the University of Technology Sydney Animal Ethics Committee.

## Author contributions

NS: all animal and experiments, data analysis, and manuscript preparation. DG: animal experiments and data analysis. MBS: access to RAGE knock-out mice, experiment design, data interpretation, and manuscript preparation. SMG: supervision of animal and laboratory-based experiments, experiment design, data analysis, data interpretation, and manuscript preparation. All authors read and approved the final manuscript.
