# Supplementary material for: RAGE and its ligand amyloid beta promote retinal ganglion cell loss following ischemia-reperfusion injury
Source: Front Cell Neurosci. 2023 Apr 12;17:1156084. doi: 10.3389/fncel.2023.1156084 (PMC10130520; doi:10.3389/fncel.2023.1156084)
Supplement: Supplementary file 1 [file Data_Sheet_1.docx]

**Supplementary Data**


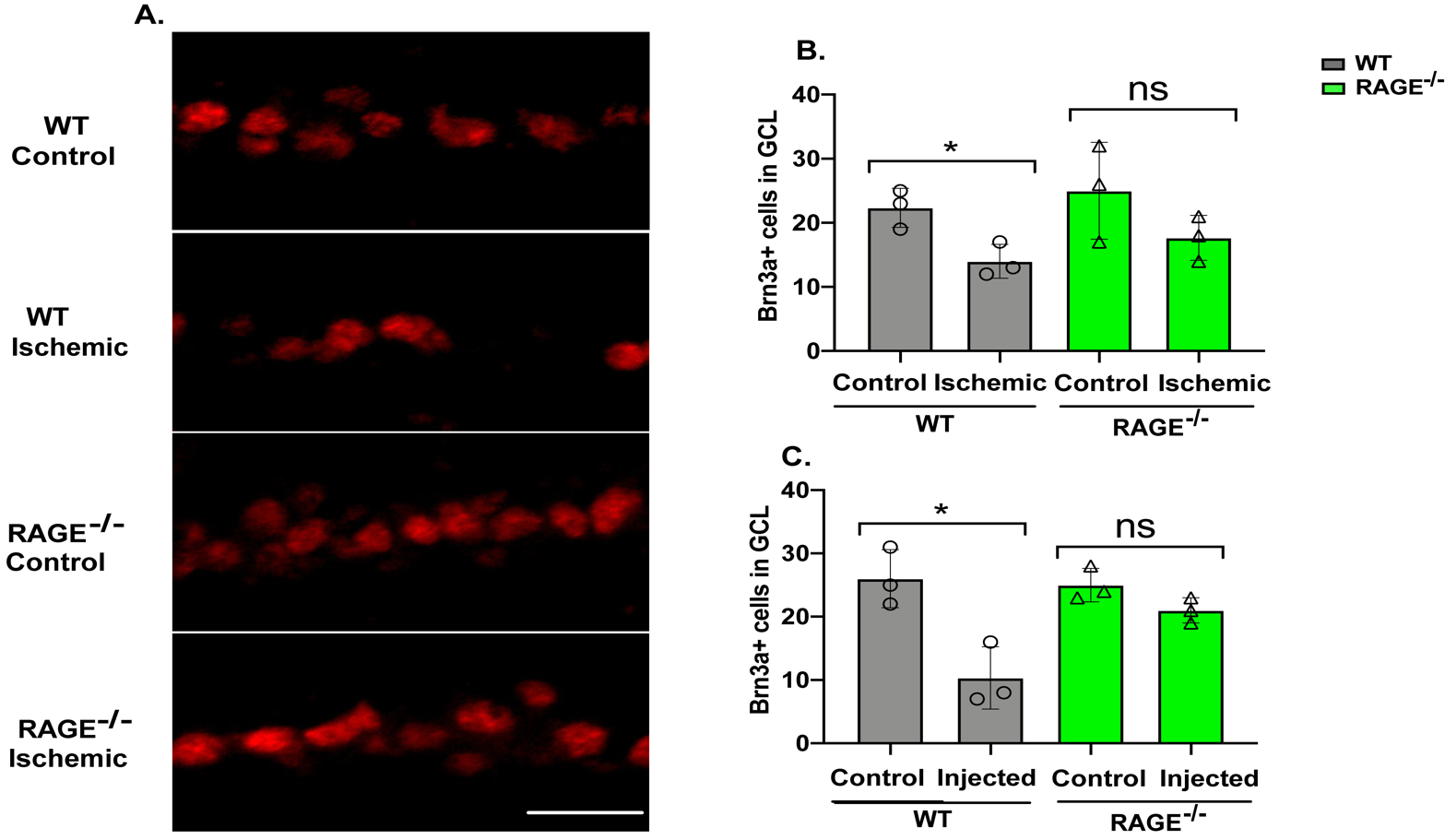


**Figure S1. RAGE^–/–^ mice are protected against RGC loss in ischemia and Aß injected groups***.* (A) Immunostaining represents RGCs using the Brn3a (red) marker in the ischemic and control group of WT and RAGE^–/–^ retinas (n=3). (B) Comparison of the alterations of RGC numbers in WT and RAGE^–/–^ mice after ischemia. A significant decline in RGC numbers was observed in WT but not RAGE^–/–^ mice. (C) Comparison of the alterations of RGC numbers in WT and RAGE^–/–^ groups 7 days post- Aß injection (n=3). A significant decline in RGC numbers were confirmed in WT but not RAGE^–/–^ mice. *p=0.02, ns: no significance unpaired t-test. The scale bar is 50 µm.


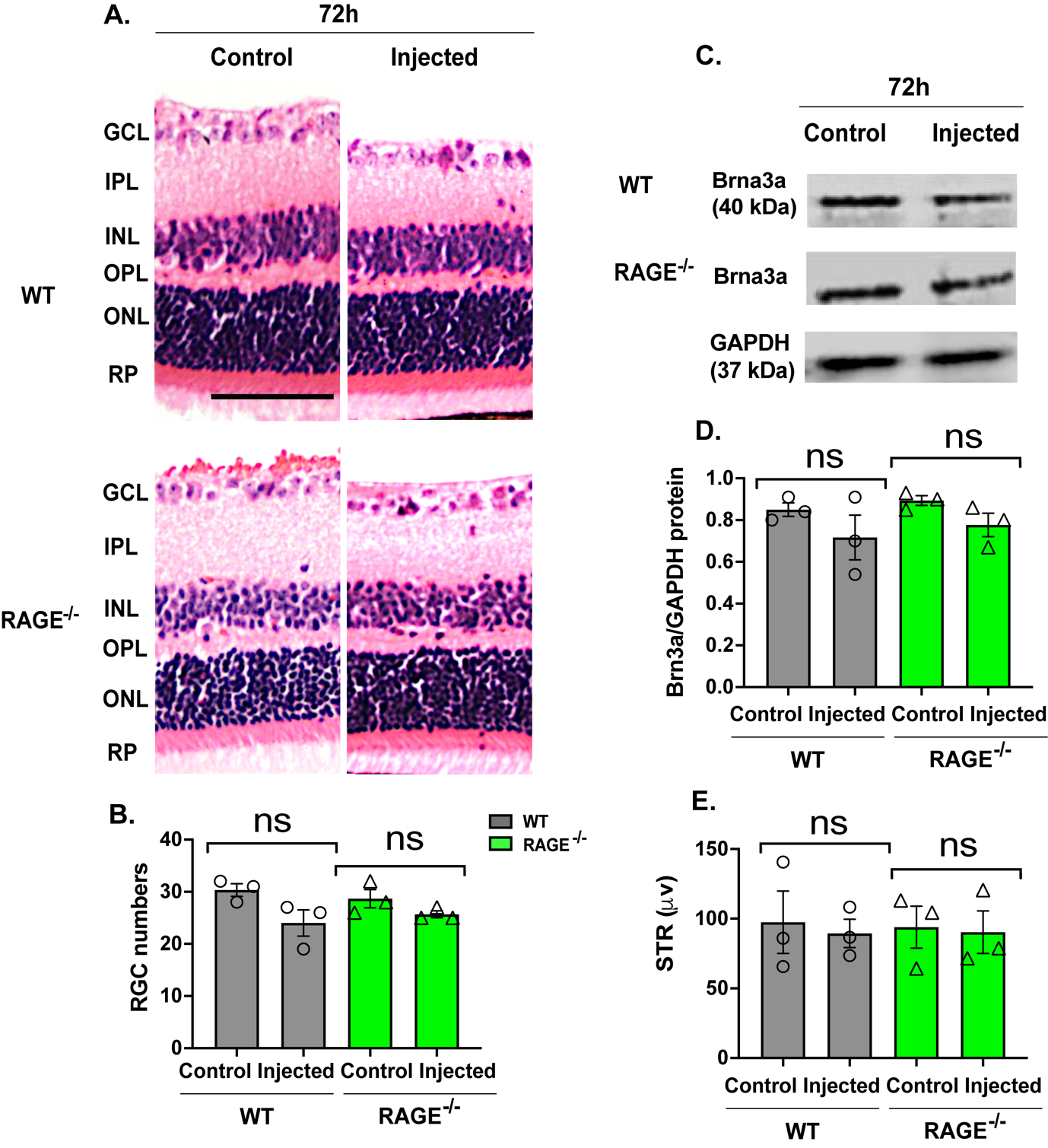


**Figure S2. Intravitreal injection of Aß leads to nonsignificant RGC loss and inner in WT and not RAGE^–/–^ mice 72 h post Aß injection.** (A) H&E staining of WT and RAGE^–/–^ retina, 72h after Aß injection (n=3). non-significant RGC loss in both WT and RAGE^–/–^ retina. RGCs were measured manually around the optic nerve head in increments of 300µm. (B) Comparison of the alterations of RGC numbers in WT and RAGE^–/–^ mice after Aß injection. Using H&E staining, a non-significant decline in RGC numbers was observed in both WT and RAGE^–/–^ mice (n=3). (C) Western blotting of WT and RAGE^–/–^ retinas after intravitreal injection of Aß. GAPDH was used as a control (n=3). (D) A non-significant Brn3a downregulation was observed in WT, 72h after Aß injection. Brn3a downregulation in Aß injected eyes of each mouse was normalized as Brn3a/GAPDH (n=3) (E) Graph representing pSTR analysis of WT and RAGE^–/–^ (n=3), ns: no significance, unpaired t-test. The scale bar is 100 µm.
